# Modeling the Interplay between Photosynthesis, CO_2_ Fixation, and the Quinone Pool in a Purple Non-Sulfur Bacterium

**DOI:** 10.1038/s41598-019-49079-z

**Published:** 2019-09-02

**Authors:** Adil Alsiyabi, Cheryl M. Immethun, Rajib Saha

**Affiliations:** Department of Chemical and Biomolecular Engineering, University of Nebraska-Lincoln, Lincoln, NE USA

**Keywords:** Biochemical networks, Systems analysis

## Abstract

*Rhodopseudomonas palustris* CGA009 is a purple non-sulfur bacterium that can fix carbon dioxide (CO_2_) and nitrogen or break down organic compounds for its carbon and nitrogen requirements. Light, inorganic, and organic compounds can all be used for its source of energy. Excess electrons produced during its metabolic processes can be exploited to produce hydrogen gas or biodegradable polyesters. A genome-scale metabolic model of the bacterium was reconstructed to study the interactions between photosynthesis, CO_2_ fixation, and the redox state of the quinone pool. A comparison of model-predicted flux values with available Metabolic Flux Analysis (MFA) fluxes yielded predicted errors of 5–19% across four different growth substrates. The model predicted the presence of an unidentified sink responsible for the oxidation of excess quinols generated by the TCA cycle. Furthermore, light-dependent energy production was found to be highly dependent on the quinol oxidation rate. Finally, the extent of CO_2_ fixation was predicted to be dependent on the amount of ATP generated through the electron transport chain, with excess ATP going toward the energy-demanding Calvin-Benson-Bassham (CBB) pathway. Based on this analysis, it is hypothesized that the quinone redox state acts as a feed-forward controller of the CBB pathway, signaling the amount of ATP available.

## Introduction

Purple non-sulfur bacteria (PNSB) are considered to be among the most metabolically versatile groups of bacteria^1,2^. Within this class, *Rhodopseudomonas palustris* CGA009 (hereafter *R*. *palustris*) demonstrates this elasticity through its ability to survive in a myriad of diverse environmental conditions^3^. It can grow either aerobically or anaerobically, utilize organic (heterotrophic) or inorganic (autotrophic) carbon sources, and exploit light to obtain energy when growing anaerobically^3^. Several interesting features have been observed in this bacterium, such as its consumption of fatty acids, dicarboxylic acids, and aromatic compounds including lignin breakdown products (LBPs)^4–6^. It is also one of two known bacteria that can express three unique nitrogenases, each with a different transition-metal cofactor^7^. Furthermore, this metabolically versatile strain’s genome encodes the aerobic and anaerobic pathways for three of the four known strategies that microbes use to break down aromatic compounds, such as LBPs^8^. Harnessing *R*. *palustris*’ unique metabolic versatilities for the conversion of plant biomass to value-added products, such as polyhydroxybutyrate (PHB)^9^, n-butanol^10^, and hydrogen^11,12^, has garnered increasing interest. However, lack of a systems-level understanding of how the bacterium’s complex web of metabolic modules operates in response to environmental changes is hindering the development of the PNSB as a biochemical chassis.

Several studies conducted on *R*. *palustris* showed that in addition to the Calvin-Benson-Bassham (CBB) cycle’s role of carbon assimilation during autotrophic growth, the pathway plays a major role in maintaining redox balance under heterotrophic conditions^10,12–14^. It was shown that heterotrophic growth of the PNSB on substrates that are more reduced than biomass, such as LBPs, is dependent on the availability of an electron sink^13^. CO_2_-fixation using the enzyme ribulose-1,5-biphosphate carboxylase/oxygenase (RuBisCO), nitrogen-fixation through the enzyme nitrogenase^12^, and supplementation with an electron acceptor (e.g., trimethylamine-N-oxide (TMAO))^15^ all prevent the inhibitory accumulation of excess reducing agents. Therefore, the use of CO_2_ as a redox balancing strategy for the conversion of plant biomass to value-added products is an attractive approach that could increase profitability while improving sustainability. However, the complex interplay between the electrons supplied by the catabolism of different carbon sources, CO_2_ fixation, and the cyclic electron flow during photosynthesis is not fully understood, thus diminishing the ability to engineer this promising bacterium.

A Genome-Scale Metabolic Model (GSMM) provides a mathematical representation of an organism’s metabolic functionalities^16,17^ by translating the repertoire of biochemical transformations into a stoichiometric matrix^18^. Due to the underdetermined nature of metabolic networks, optimization tools are used to predict reaction rates for a pre-specified objective function, such as the maximization of biomass^19^. One of the most common optimization tools used to model metabolism is Flux Balance Analysis (FBA). FBA performs a pseudo-steady state mass balance for each metabolite in the network to predict the maximum growth rate and corresponding reaction fluxes during the cell’s exponential growth phase^20–24^. Due to the high dimensionality of the network, other tools such as Flux Variability Analysis (FVA) are used to determine the sensitivity of growth rate as a function of each reaction flux^25^. Finally, a modified FBA formulation can be used to predict the set of essential genes under a specified growth condition^26^. Thus far, a limited number of small-scale metabolic reconstructions have been developed for PNSB, examining either the central carbon metabolism^27^ or the electron transport chain^28^. However, these models are limited in scope, as they consider less than 4% of the organism’s metabolic functionality and are therefore incapable of capturing system-wide interactions between different metabolic modules. Very recently, a GSMM of the bacterium was reconstructed and used to test an array of cellular objectives during phototrophic growth. Anaerobic growth on acetate, benzoate, and 4-hydroxybenzoate was simulated using eight different biologically relevant objective functions^29^. The model predicted that the organism primarily optimized for growth, ATP production, and metabolic efficiency^29^. However, the model could be improved further by integrating recently annotated metabolic pathways for lignin monomer degradation^30^, as well as making use of experimental data on gene essentiality^31^ and metabolic flux analysis for growth under different carbon sources^13,14^ to validate and refine the network.

In this work, a GSMM of *R*. *palustris* (*iRpa*940) was constructed to model the bacterium’s metabolic functionality under different environmental conditions. The model was used to simulate growth on different carbon sources and showed excellent agreement with experimentally measured fluxes^13,14^. Gene essentiality analysis was also performed for aerobic and anaerobic growth on acetate. The predicted essential genes were compared with available trans-mutagenesis data^31^, and an accuracy of 84% was achieved. After the model indicated the presence of an unidentified quinol sink, *in silico* simulations were combined with published *in vivo* flux measurements^13,14^ to study the effect (and the extent) of the quinone redox state on cellular growth, electron transport rate, and CO_2_ fixation. It was observed that an increase in the quinol oxidation rate resulted in an increase in the electron transport rate, and therefore ATP generation. These results suggest that redox state acts as a feed-forward controller of the highly energy-demanding CBB cycle by regulating the rate of light-generated ATP. Overall, an understanding of the metabolic control points of this interconnected system constitutes the first step towards engineering strains capable of more efficiently harnessing photosynthetic energy and rerouting this energy towards bio-production and lignin valorization.

## Methods

### Model reconstruction

A draft model was first generated in KBase^32^ based on *R*. *palustris*’ genome (downloaded from the NCBI database on 04/12/2018). KBase uses annotated features in the genome to construct a list of reactions associated with genes in the organism. Previously published work of the bacterium’s metabolic network^27^ was used to manually curate pathways from the central carbon metabolism and to ensure correct cofactor usage and gene association. This resulted in an expanded network of high-confidence reactions, all associated with genes in *R*. *palustris*. Experimentally measured concentrations of biomass components are available for *R*. *palustris* when grown on acetate^13^, and were used to develop the biomass equation (see Supplementary File 1). To minimize the addition of low-confidence reactions during gap-filling, the process was broken down into two steps. First, a subset of high-confidence reactions from a recently published genome-scale model of *R*. *palustris*^29^ was added to the draft model. Here, high-confidence reactions are defined to be the reactions that are associated with at least one published source of annotation. At the end of this step, the majority of the gaps in the network that precluded the production of biomass existed in partially incomplete linear pathways. Therefore, the ModelSEED database^33^ was used to fill the gaps in the network, and a biomass producing model was generated in KBase^32^. In addition, annotated metabolic pathways for the breakdown of multiple aromatic compounds including lignin breakdown products were found in literature^30^ and in organism-specific biochemical databases^34,35^, and were subsequently added to the model (see Fig. S1 in Supplementary File 2). Finally, annotated *R*. *palustris* genes were mined from three databases (KEGG^34^, BioCyc^35^, and UniProt^36^) to validate the Gene-Protein-Reaction (GPR) associations established in the model and to include GPR relationships for reactions added during the gap-filling process (see Supplementary File 3).

### Model simulations

Parsimonious Flux Balance Analysis (pFBA)^37^ was used to simulate growth under different environmental conditions. pFBA is analogous to FBA but adds a second objective that minimizes the sum of all reaction fluxes. The two objectives were reformulated into one function through objective tilting^38^ as displayed below.

1 $$\begin{array}{c}Maximize\,{v}_{biomass}-0.0001\sum _{j\in J-{v}_{biomass}}{v}_{j}\\ subject\,to\\ \sum _{j\in J}{S}_{ij}\cdot {v}_{j}=0\,{\rm{\forall }}i\in I\end{array}$$

2 $$L{B}_{j}\le {v}_{j}\le U{B}_{j}\,{\rm{\forall }}j\in J$$

where *I* and *J* are the sets of metabolites and reactions in the model, respectively. *S*_*ij*_ is the stoichiometric coefficient of metabolite *i* in reaction *j* and *v*_*j*_ is the flux value of reaction *j*. Parameters *LB*_*j*_ and *UB*_*j*_ denote the minimum and maximum allowable fluxes for reaction *j*, respectively. *v*_*biomass*_ is the flux of the biomass reaction which mimics the cellular growth rate.

### Model validation

Metabolic Flux Analysis^39,40^ (MFA) measurements for anaerobic growth on acetate^13^, fumarate^14^, succinate^14^, and butyrate^14^ were compared with model predicted fluxes. Model accuracy for each growth condition was calculated by taking the sum of percent errors between pFBA-predicted and MFA values (see Supplementary File 4 for an example). In addition, *R*. *palustris’* essential genes, determined experimentally for aerobic growth on acetate^31^, were used to validate the essential genes predicted by the model. Gene essentiality was predicted in the model by sequentially knocking out each reaction and determining the resulting effect on the biomass reaction rate^26^. If a reaction knockout resulted in a predicted growth rate that was less than 10% of the wild type growth rate, the reaction was considered essential^41,42^. Reaction GPRs were then used to map the list of essential reactions to essential genes. Finally, the list of experimentally determined essential metabolic genes^31^ were compared with model predicted essential genes to determine the specificity and sensitivity of the predictions (see Supplementary File 5).

## Results and Discussion

### Model Reconstruction and validation

**Figure 1:**
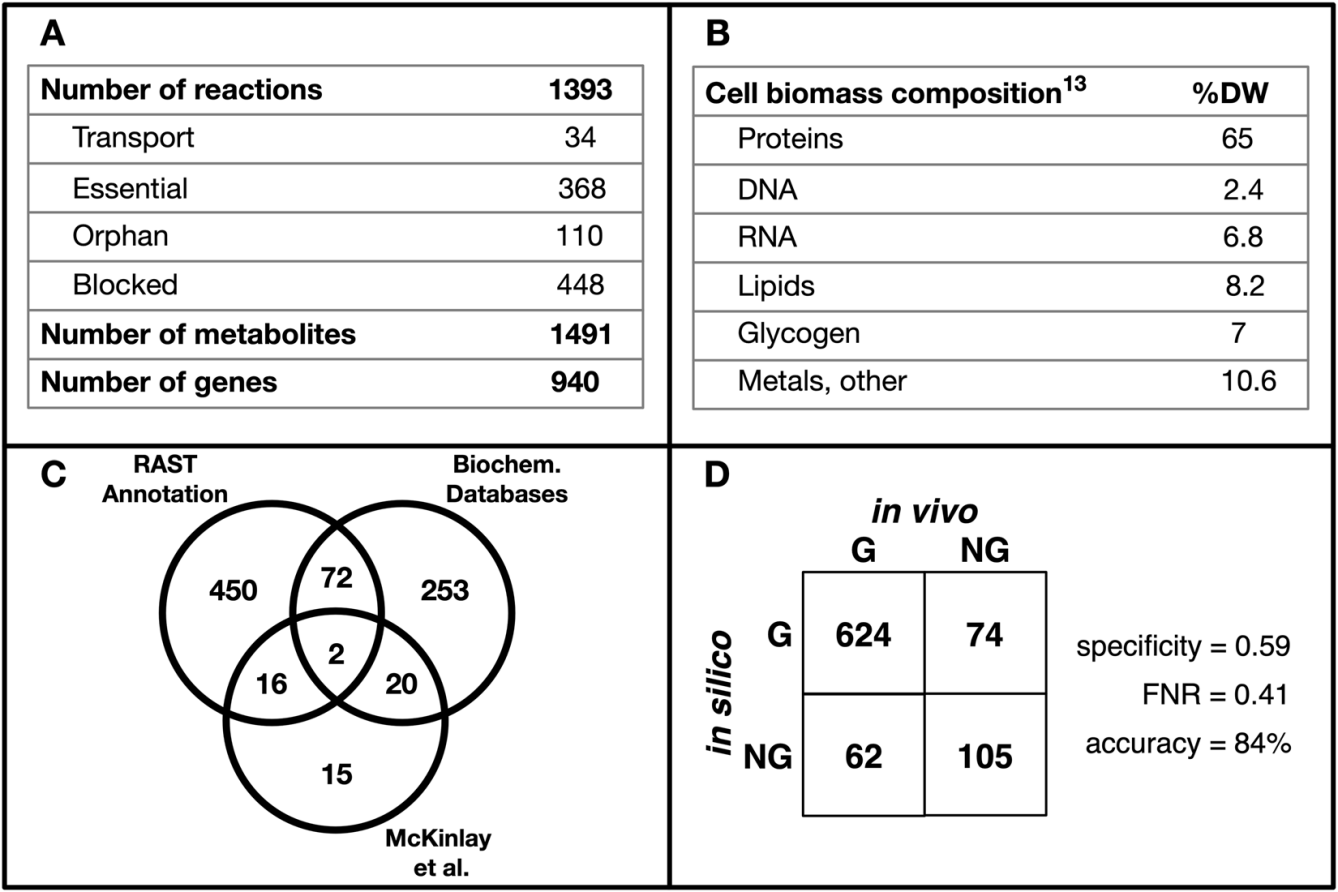
Summary of the *iRpa*940 model statistics and validation. (**A**) Overall model statistics. **(B)** Model biomass component compositions. **(C)** Sources of gene annotation. **(D)** Gene essentiality analysis results. G: Growth (non-essential gene), NG: No Growth (essential gene).

A summary of the *iRpa*940 model’s major statistics is shown in Fig. 1A. Overall, the 940 genes associated with 1393 model reactions account for 62% of the genes involved in energy metabolism, biosynthesis, carbon & nitrogen metabolism, and cellular processes in *R*. *palustris’* genome^3^. Figure 1B shows the relative molar abundance of each macromolecular class in *R*. palustris^13^. This data was used to calculate the stoichiometric coefficients of components in the model’s biomass equation (see Methods). Thus, an initial high-confidence model containing 540 genes and 915 reactions with no orphan reactions was constructed. The gap-filling procedure was carried out next in KBase^32^ using reactions from the ModelSEED database^33^. Out of the 478 reactions added during gap-filling, 368 were annotated using information from organism-specific databases (see Methods). A breakdown of the number of GPR relationships established from each annotation source is shown in Fig. 1C. This resulted in the addition of 328 annotated and 110 unannotated (orphan) reactions. The inclusion of these reactions was necessary to ensure biomass production.

pFBA was used to simulate growth on a number of different carbon sources, including carboxylic acids (acetate, fumarate, succinate and butyrate) and lignin monomers. pFBA is analogous to FBA but adds an outer objective that minimizes the sum of all reaction fluxes (see Methods). This is justified by the assumption that cells synthesize the minimum amount of cellular machinery required to maintain the maximal growth rate^37^. Simulating growth using pFBA has two main advantages over FBA. First, pFBA avoids unrealistic flux predictions for reactions participating in thermodynamically infeasible cycles (TICs)^43^. TICs are usually removed from GSMMs to avoid false predictions; however, when analyzing highly connected networks like that of *R*. *palustris*, removing these cycles can lead to the model missing certain functionalities and metabolic modes utilized by the organism. pFBA avoids these false predictions by the additional constraint that reaction fluxes should be minimized. Second, the pFBA formulation results in a significantly reduced set of optimal solutions compared to FBA. Flux Balance Analysis usually results in a large number of alternate optimal solutions (especially in highly connected networks), most of which are not biologically relevant, and can therefore lead to false conclusions^44^. pFBA’s additional objective greatly restricts the solution space and leads to more biologically insightful conclusions^37^.

*In silico* gene essentiality analysis identified 368 essential reactions, out of which 249 were associated with gene annotations in the model. These essential reactions were then compared with *in vivo* gene essentiality data for aerobic growth on acetate^31^ to check the model accuracy (Fig. 1D). The calculated sensitivity and false negative rate (FNR) were consistent with recently published GSMMs^45,46^. Moreover, given that this is a non-model organism with no well-characterized close relatives, high-confidence annotation was not available for the less-studied pathways. Therefore, an automated pipeline like GrowMatch^47^ could not be implemented with justifiable accuracy to further improve essentiality predictions.

### The effect of the quinone pool on light uptake, carbon dioxide fixation, and growth

During initial phototrophic growth simulations, growth on any of the four carbon sources (acetate, fumarate, succinate, and butyrate) was observed to be hindered due to the accumulation of excess quinols formed in the TCA cycle. Flux analysis of the electron transport chain (ETC) revealed that the rate of quinol oxidization through the cytochrome bc1 complex was equivalent to the rate of quinone reduction in the Reaction center (RC). This result is consistent with previous studies in PNSB^28^, and is necessary for steady-state flow of electrons through the cyclical chain. Furthermore, previous studies on the activity of the ETC concluded that the thermodynamically unfavorable process of reverse electron transfer through NADH dehydrogenase had very low activity compared to the rate through the RC^28,48^. Therefore, this reaction could not account for the oxidation of the excess quinols produced in the TCA cycle. Since no other high-confidence reaction was found to consume quinols in *R*. *palustris*, a quinol “sink reaction” was added to the *iRpa*940 model. Sink reactions are often incorporated into metabolic models when a metabolite is known to be produced during metabolism but for which no means of consumption have been identified^49^, or to describe the accumulation of a storage compound^49^ (e.g. glycogen). Furthermore, recent experimental work with *R*. *palustris* TIE-1 reported the presence of an unidentified quinol-oxidizing reaction that had not been accounted for previously^48^, giving further support to this prediction.

**Figure 2:**
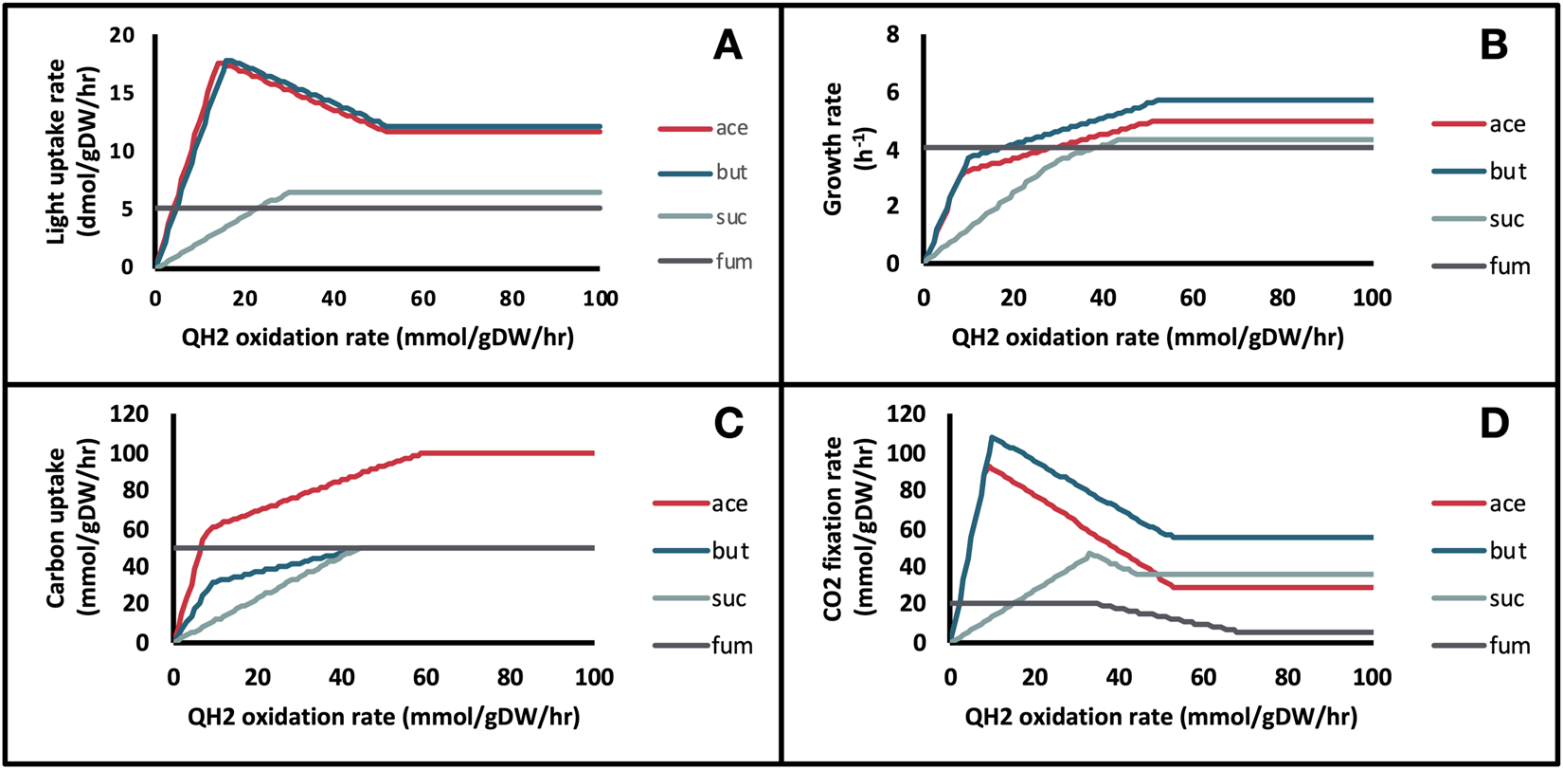
Effect of the Quinol sink rate on: (**A**) Light uptake rate, (**B**) Growth rate, (**C**) Carbon source uptake rate, and (**D**) Carbon fixation rate for growth on four carbon sources. ace: acetate, but: butyrate, suc: succinate, fum: fumarate.

**Figure 3:**
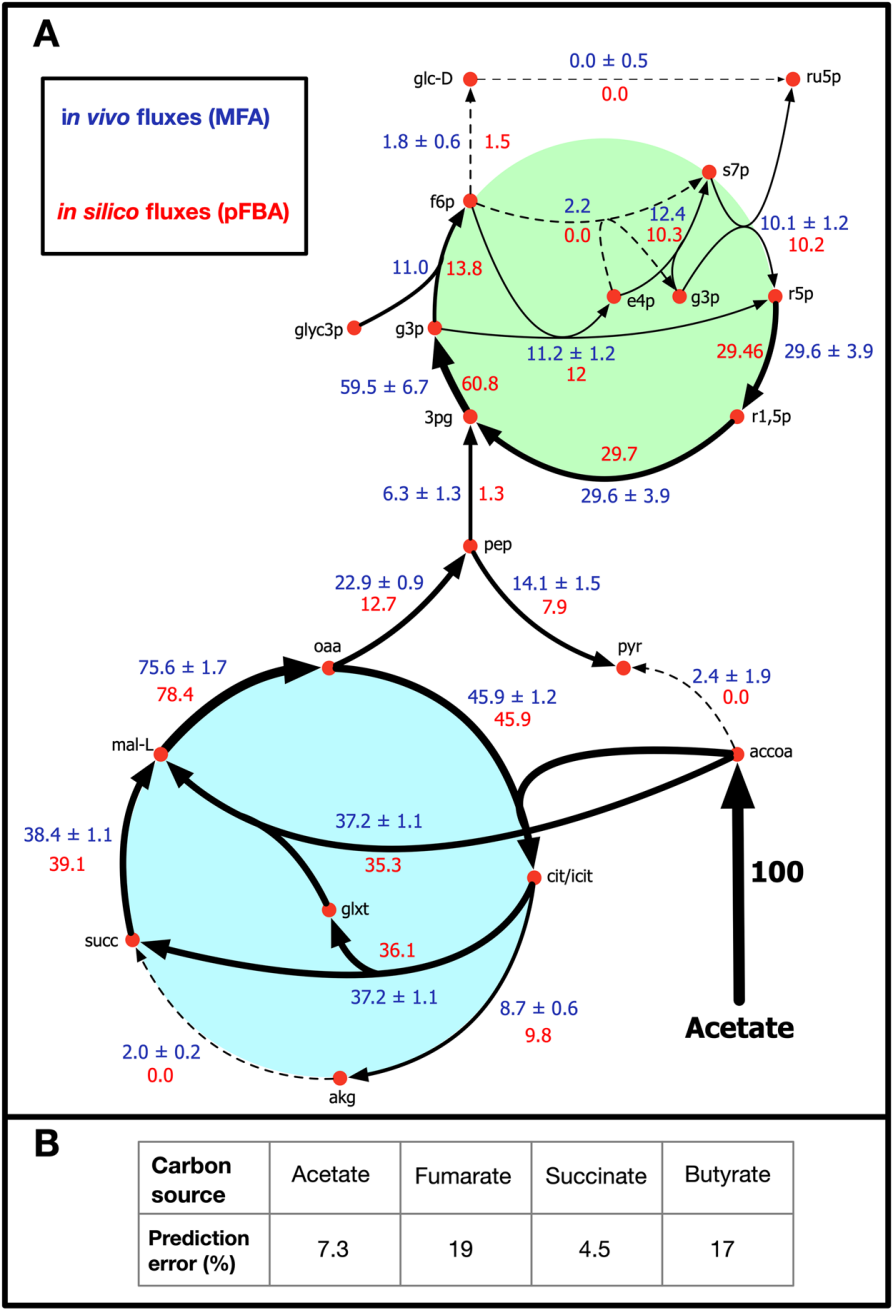
Comparison of model-predicted vs MFA-generated flux values for reactions involved in central carbon metabolism. (**A**) Metabolic flux map showing reaction rates for growth on acetate **(B)** Percentage error between model predictions and MFA flux values for growth on four carbon sources.

pFBA simulations were conducted under different quinol sink rates to qualitatively predict how changes in the quinone redox state affected the rest of the metabolic network. The quinol sink reaction was treated as a parameter in the model and pFBA simulations were conducted at varying quinol oxidation (sink) rates to determine how light uptake (*i*.*e*. Electron Transport Rate or ETR), growth, and CO_2_ fixation are affected by changes in the quinone redox state (Fig. 2). Carbon uptake was restricted to a maximum value of 100 mmol/gDW/hr for acetate and 50 mmol/gDW/hr for fumarate, succinate, and butyrate to ensure the same number of carbons were being taken up. MFA values were scaled to the same carbon uptake rates^13,14^. For growth on butyrate, the supplementation of CO_2_ is required for growth, as the substrate is more reduced than biomass and requires an electron sink^14^. The media was supplied with CO_2_ at a maximum uptake rate of 32.1 mmol/gDW/hr to match MFA observations. Since steady-state GSMMs cannot capture metabolite concentrations, the redox state cannot be quantified directly. Instead, the qualitative behavior of the redox state was predicted by varying the rate of the quinol sink. As the quinol oxidation rate increases, the quinone pool becomes more oxidized. Using experimental MFA data^13,14^, the quinol oxidation rate was predicted for each of the four substrates (Table 1). These values were calculated by minimizing the sum of errors between the *in silico-*generated pFBA fluxes and the MFA flux values. The table also shows the quinone reduction rate through the TCA cycle for each carbon source. The percentage of CO_2_ fixed was defined as the rate of CO_2_ fixation divided by the total rate of CO_2_ produced metabolically. Figure 3 shows the resulting flux predictions obtained at the predicted quinol oxidation rates for growth on acetate (Fig. 3A), and the calculated percent errors of these predictions for each carbon substrate (Fig. 3B). A comparison of flux predictions with MFA values for the other three carbon sources is provided in Supplementary File 2 (see Figs S2–S4).

**Table 1:** Predicted reaction rates for growth on four different carbon sources.

Carbon source	QH_2_ oxidation rate (mmol/gDW/hr)	Q reduction rate^a^ (mmol/gDW/hr)	Electron transport rate (dmol/gDW/hr)	CO_2_ fixation rate (mmol/gDW/hr)	% CO_2_ fixed^b^	Net CO_2_ excretion rate (mmol/gDW/hr)
Acetate	52.5	39.1	5.3	29.7	73.2	10.9
Butyrate	54.9	37.4	5.4	57.8	—	−18.6^c^
Succinate	49.8	49.2	3.0	35.6	50.6	34.8
Fumarate	0	0	2.3	17.3	25.1	51.5

^**a**^The rate of quinone reduction in the TCA cycle.

^**b**^The rate of CO_2_ fixation divided by the rate of total CO_2_ produced.

^**c**^CO_2_ was supplied in the media during growth on butyrate.

For growth on acetate and butyrate, light uptake (*i*.*e*. ETR) showed two distinct regions based on the extent of quinol oxidation (Fig. 2). Under low oxidation rates, flux through the quinol-producing succinate dehydrogenase reaction was avoided by using the glyoxylate shunt and subsequently the CBB cycle. Therefore, both light uptake and CO_2_ fixation increased rapidly in this region. In the second region, at high quinol oxidation rates, flux shifted toward the oxidative TCA cycle. Therefore, in this region, both the Electron Transport Chain (ETC) activity and the rate of CO_2_ fixation decreased with increasing quinol oxidation. Furthermore, as can be seen from Table 1, the ratio of quinol oxidation rate to quinone reduction rate was similar for both carbon sources. Due to the supplementation of CO_2_ during growth on butyrate, the percentage of CO_2_ fixation could not be calculated. During growth on succinate, the production of quinols through succinate dehydrogenase could not be avoided, therefore light uptake rate increased linearly with the rate of quinol oxidation. Moreover, the rates of quinol oxidation and quinone reduction were equivalent, indicating that the quinone pool was more reduced when compared to the redox state during growth on acetate and butyrate. This led to a reduced electron flow through the ETC, and subsequently lower ATP generation. Finally, the model predicted that during growth on the highly oxidized (compared to cell biomass) carbon source fumarate, the rate of the quinol sink did not affect the flux distribution.

**Figure 4:**
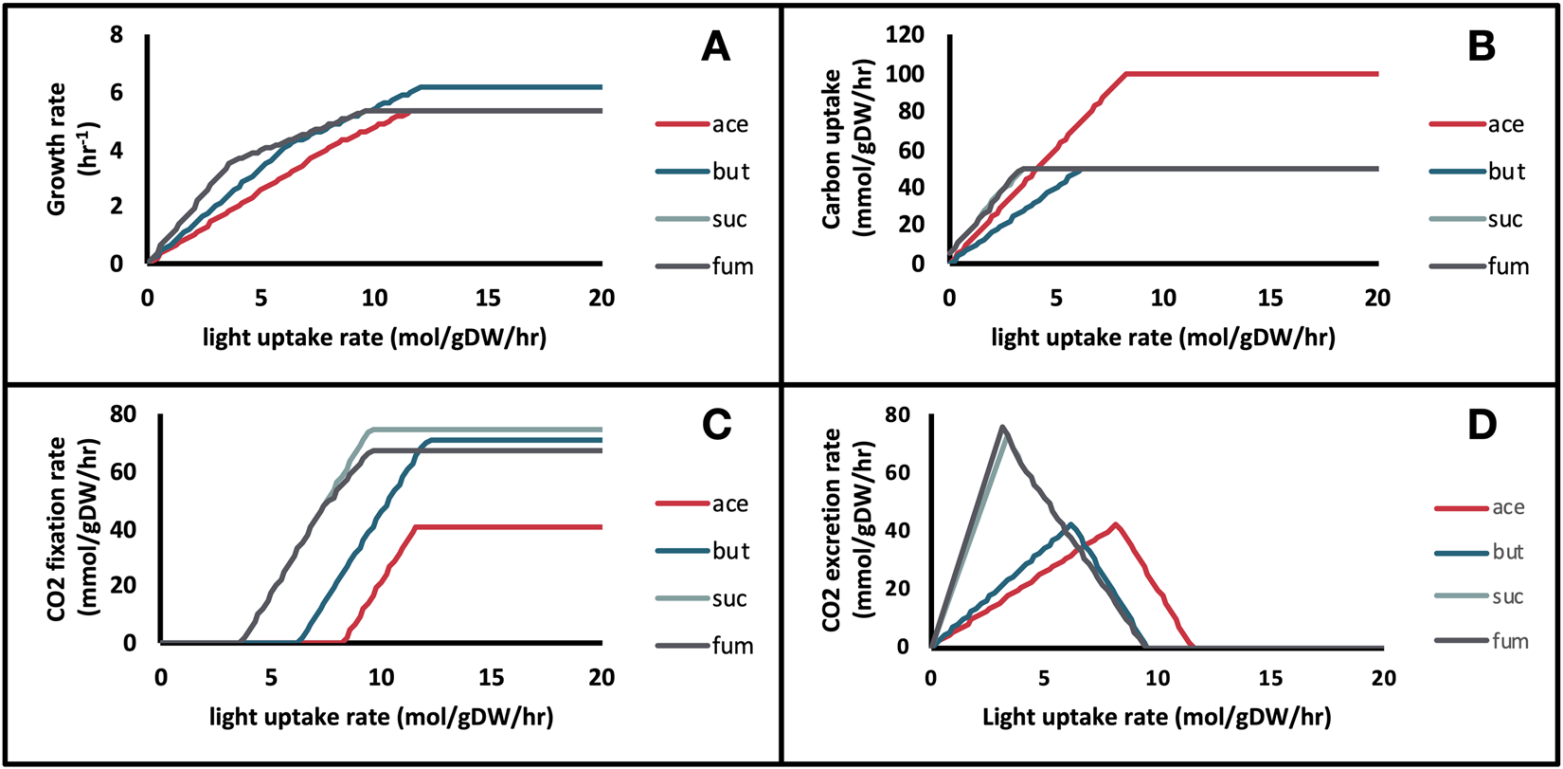
Effect of the light uptake rate on (**A**) Growth rate, (**B**) Carbon source uptake rate, (**C**) Carbon fixation rate, and (**D**) Carbon dioxide excretion rate for growth on four carbon sources. ace: acetate, but: butyrate, suc: succinate, fum: fumarate. In A, B, and D, the lines for succinate and fumarate lie on top of each other.

A similar parameter sampling procedure was performed to determine the effect of light uptake on growth. Light uptake rate was set as a parameter and the quinol oxidation rate was fixed to the value predicted based on MFA fluxes (Fig. 4). Again, there were two distinct growth regions: (i) a low-light (LL) energy-limited region, and (ii) a high-light (HL) carbon-limited region. In the LL region, growth was highly dependent on the amount of light available and the model predicted that all of the ATP produced was used to convert the carbon source into biomass precursors. Therefore, no ATP remained for the energy-intensive CBB pathway. In the HL region, when the maximum substrate uptake rate was reached, the carbon source could not be incorporated any faster. The additional energy produced from light was then directed towards CO_2_ fixation. Although the model predicted that the rate of CO_2_ fixation increased linearly with light uptake rate, kinetic and thermodynamic constrains on the highly inefficient CO_2_-fixing RuBisCO enzyme^50^ hinders this process at high light uptake.

### Proposed mechanism for the interplay between the quinone redox state, the electron transport rate, and CO_2_ fixation

**Figure 5:**
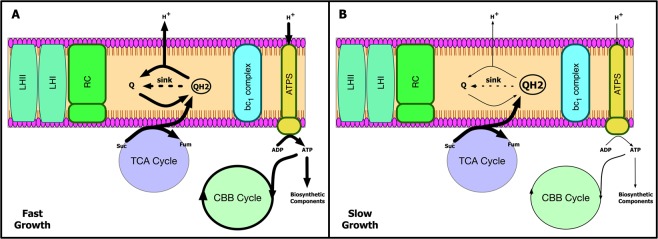
Schematic of a proposed mechanism for the interaction between the quinone redox state, electron transport rate, and carbon fixation. (**A**) High rate of quinol oxidation. (**B**) Low rate of quinol oxidation.

Based on how the quinol oxidation rate effected the light uptake and the model’s flux distribution, a mechanistic explanation of the system-wide metabolic interactions can be postulated. During steady-state operation of the cyclic ETC, the flux through the quinone reducing RC and quinol oxidizing cytochrome bc1 complex are coupled to ensure a constant rate of electron flow through the cycle^28^. Therefore, as shown in Fig. 5, increased flux through the oxidative TCA cycle leads to the accumulation of reduced quinols. This in turn leads to a restriction in the flow of electrons through the ETC and consequently in the amount of ATP produced. The CBB system thus lacks the energy required to fix CO_2_. Therefore, the quinone redox state is predicted to act as a feed-forward controller to the energetically expensive CBB pathway, indicating how much ATP is available at a given condition.

Comparison of pFBA-generated growth simulations with MFA data led to the hypothesis that an unidentified quinone oxidoreductase reaction has to occur to obtain the observed flux distribution. A previous study on the PNSB *R*. *capsulatus* suggests that complex I, the NADH:quinone oxidoreductase enzyme, is responsible for the observed quinol oxidation through reverse electron flow^51^. However, the model predicted that the rate of quinol oxidation required cannot be accounted for through complex I only, which showed low activity. Furthermore, based on the high thermodynamic cost of reverse electron flow, it appears unlikely that it can account for the predicted rate of quinol oxidation^28^.

Although the source of quinol oxidation (sink) is yet to be identified, there are a number of candidate reactions that could perform this role. Primarily, the malate:quinone dehydrogenase (MDH) appears to be a potential reaction for oxidizing excess quinols. In the forward direction, this reaction converts malate into oxaloacetate and produces ubiquinol in the process. A second NAD-dependent malate dehydrogenase is also coded for by *R*. *palustris* and could perform the same function. Knocking out and over-expressing these enzymes could be employed to investigate their role in ETR, ATP production, and CO_2_ fixation.

## Conclusion

In this study, a genome-scale metabolic network (*iRpa*940) was used to propose a system-wide mechanistic model of the interactive system that includes photosynthesis, carbon dioxide fixation, and the quinone redox state. The model was validated using experimental genome essentiality data^31^ (84% accuracy) and flux measurement data^13,14^ for four carbon sources (5–19% prediction error). Model simulations predicted the presence of an unidentified quinol sink. Predictions also indicated that the extent of CO_2_ fixation is dependent on the amount of ATP present, with the quinone redox state acting as a feed-forward signal to the CBB system. Going forward, the proposed mechanism can be used to generate strategies for engineering strains capable of more efficiently harnessing photosynthetic energy, and that have the ability to reroute energy towards bio-production and lignin valorization. Future experimental work will be conducted to measure the electron transport rate, intracellular ATP concentration, and RuBisCO gene expression across different quinone redox states to strengthen the proposed hypothesis and further refine the model.

## Supplementary information

Supplementary File S1

Supplementary File S2

Supplementary File S3

Supplementary File S4

Supplementary File S5

## Data Availability

All data generated or analyzed during this study are included in this published article (and its Supplementary Information Files).
