# Supplementary material for: Modeling the Interplay between Photosynthesis, CO2 Fixation, and the Quinone Pool in a Purple Non-Sulfur Bacterium
Source: Sci Rep. 2019 Sep 2;9:12638. doi: 10.1038/s41598-019-49079-z (PMC6718658; doi:10.1038/s41598-019-49079-z)
Supplement: Supplementary file 2 — Supplementary File S2 [file 41598_2019_49079_MOESM2_ESM.pdf]

Modeling the Interplay between Photosynthesis, CO_2 Fixation, and the Quinone Pool in a Purple Non-Sulfur Bacterium

Adil Alsiyabi[1], Cheryl Immethun[1], Rajib Saha*[1]

[1]Department of Chemical and Biomolecular Engineering, University of Nebraska-Lincoln

*****Corresponding author:

Rajib Saha

Assistant Professor

Chemical and Biomolecular Engineering,

University of Nebraska-Lincoln

Lincoln, NE-68588, USA

Email: rsaha2@unl.edu

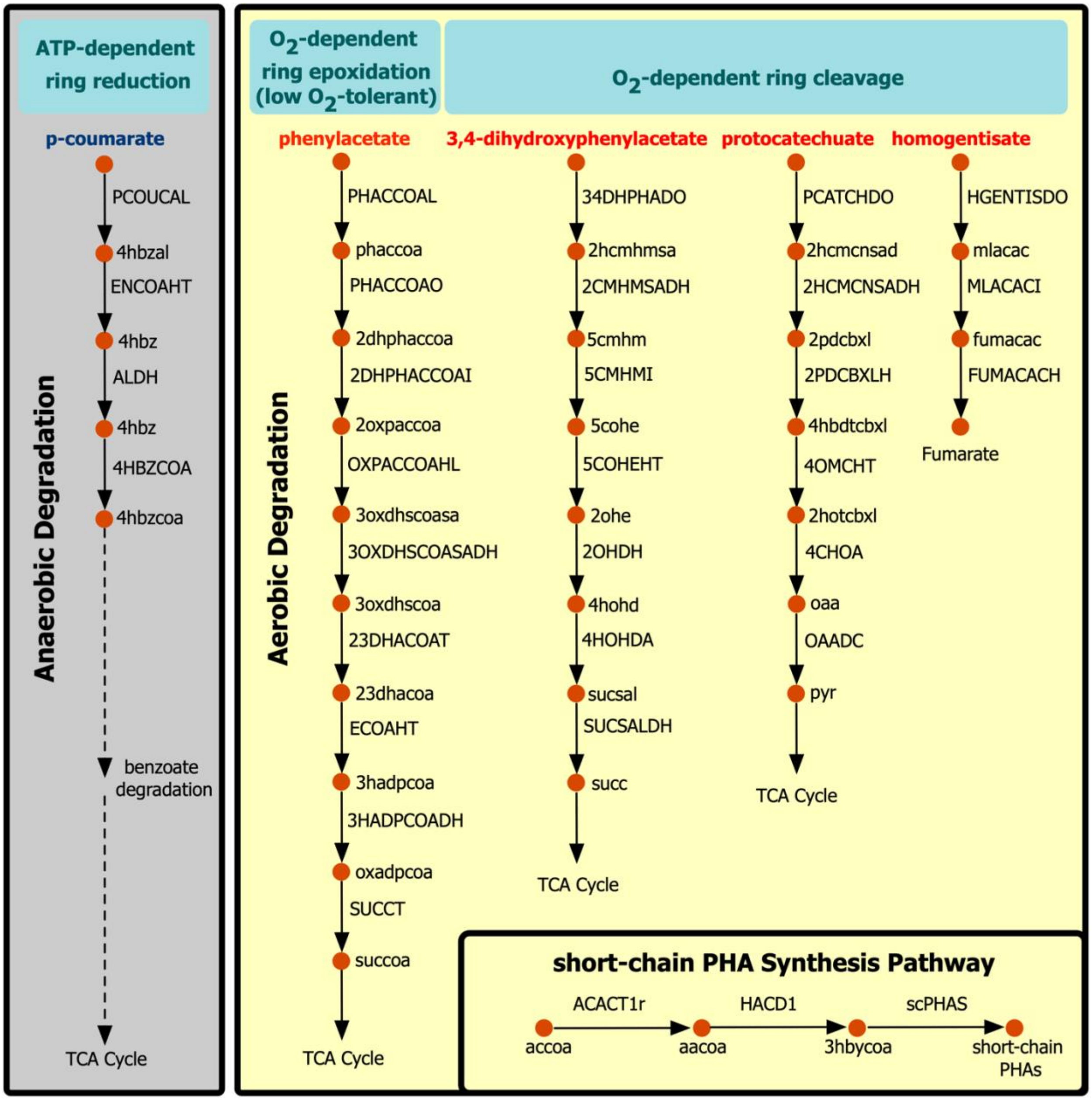

Figure S1. ***R palustris*' aromatic compound degradation and PHA synthesizing pathways.**

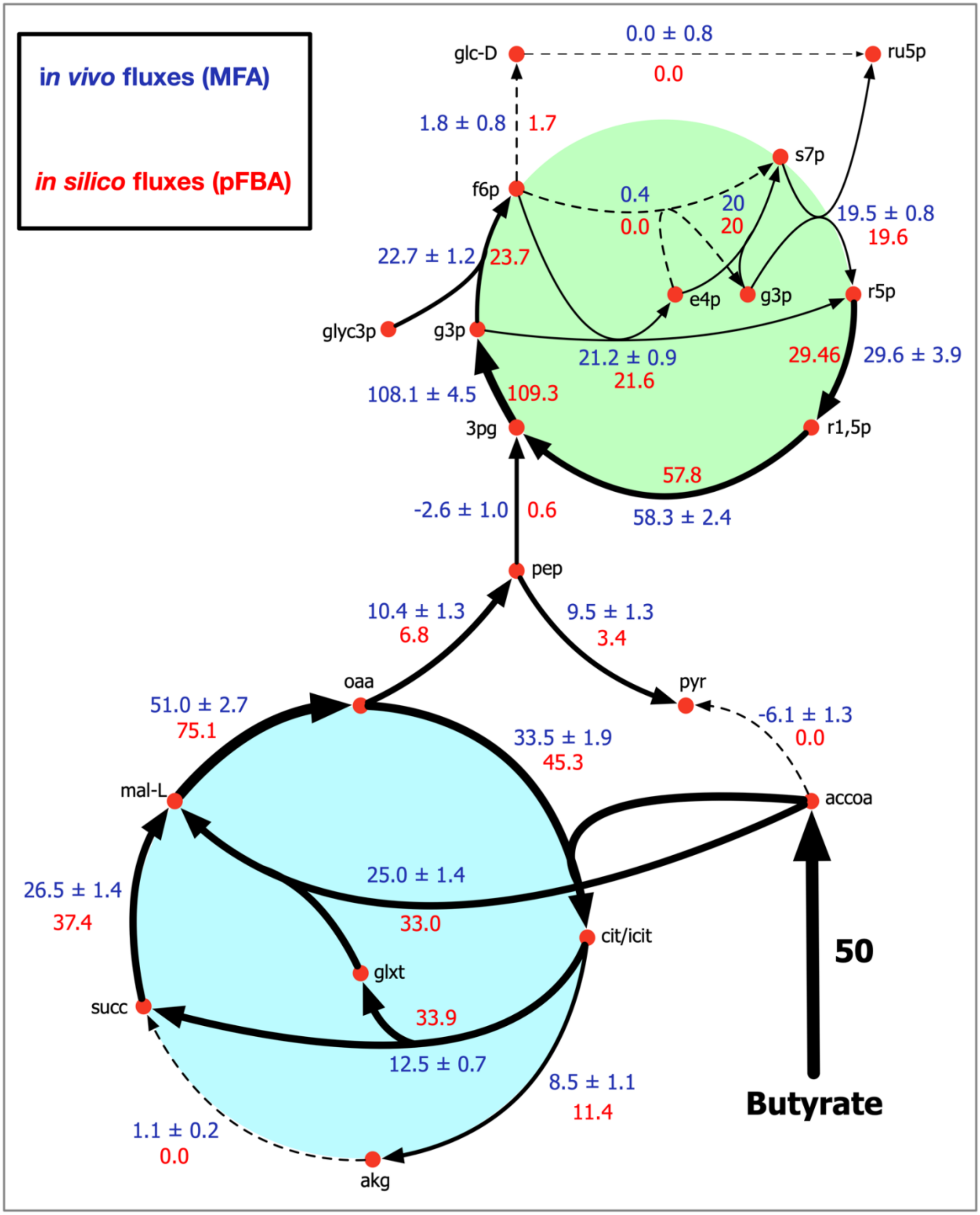

Figure S2. Metabolic flux map showing predicted pFBA and experimentally obtained MFA reaction rates for growth on butyrate.

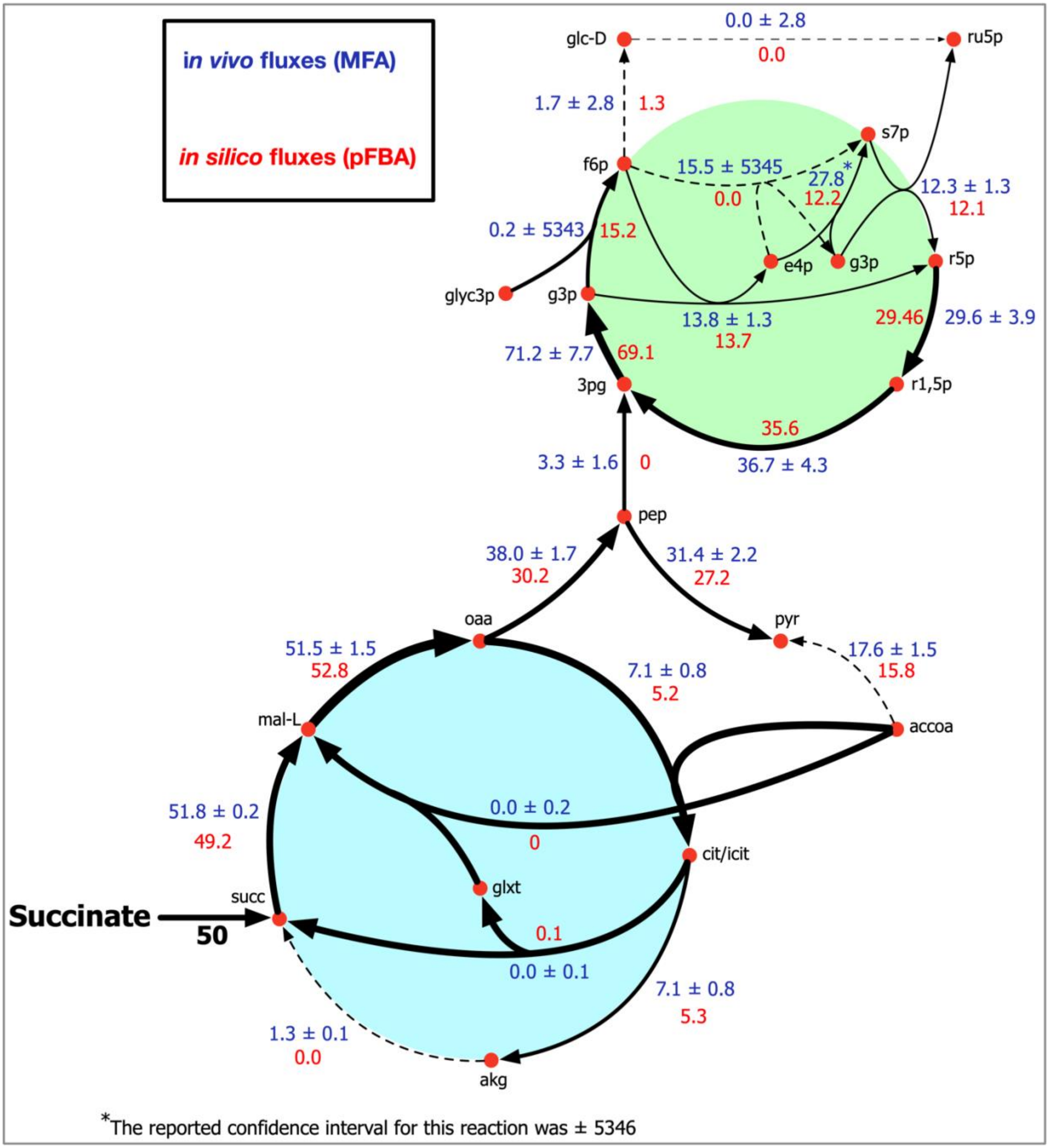

Figure S3. Metabolic flux map showing predicted pFBA and experimentally obtained MFA reaction rates for growth on succinate.

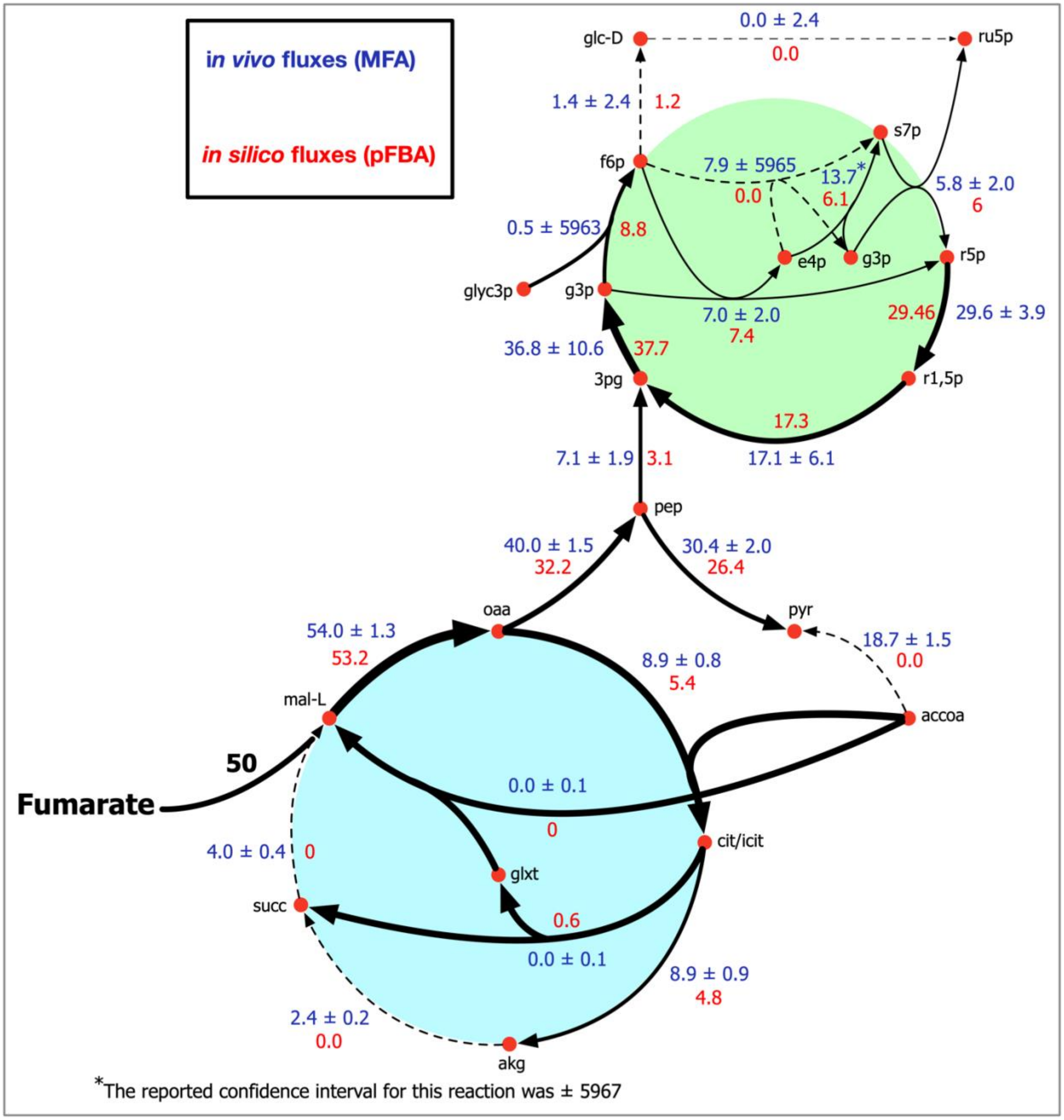

Figure S4. Metabolic flux map showing predicted pFBA and experimentally obtained MFA reaction rates for growth on fumarate.
